# City of Syracuse

**Published:** 1916-04

**Authors:** F. W. Sears

**Affiliations:** Health Officer


### City of Syracuse.

Department of Public Safety, Board of Health, replied as follows:

In response to your inquiry regarding the dismissal after contagious diseases, will state at the present time the only two diseases which we dismiss on bacteriological findings. are Diphtheria and Typhoid Fever.

Our rule on Diphtheria is after two negatives, twenty-four hours apart, for the patient, and one negative on the other members of the family. On Typhoid Fever when two negative urine and stools are found. On Scarlet Fever the minimum time limit is thirty days, but then only on inspection by our Deputy Health Officer as to the freedom from discharges from the ear and nose, or any glandular discharges, and a perfect freedom from desquamation. Chicken Pox, when there is entire freedom from crusts, and a similar regulation for Smallpox.

My opinion in the matter of the release is that the above regulations are fairly satisfactory, yet I think it would be better to have a minimum time limit of quarantine on Diphtheria, say possibly ten or twelve days. The reason for that is that it so often happens with us that after two negative cultures, we might again find a positive, and I feel it is hardly safe to allow a child the entire freedom from quarantine to mingle with other children, under say twelve days, even though we do have two negatives.

In regard to Typhoid Fever we feel that if, after the entire cessation of the febrile symptoms, we get two negatives from the stools and the urine, we are fairly safe, provided the examination of the stool is conducted say an hour after it is discharged.

I do not feel that in the case of Scarlet Fever, thirty days is an unnecessarily prolonged period, as we find in many of our cases a somewhat delayed desquamation, and, also, are not able to feel positive that we will not get some complications like ear trouble or enlarged glands.

Trusting I have made myself clear, I am, very truly yours,

F. W. SEARS, Health Officer.

